# A Lagrange-Newton Method for EIT/UT Dual-Modality Image Reconstruction †

**DOI:** 10.3390/s19091966

**Published:** 2019-04-26

**Authors:** Guanghui Liang, Shangjie Ren, Shu Zhao, Feng Dong

**Affiliations:** 1 Tianjin Key Laboratory of Process Measurement and Control, School of Electrical and Information Engineering, Tianjin University, Tianjin 300072, China; ghliang@tju.edu.cn (G.L.); fdong@tju.edu.cn (F.D.); 2 Institute of Biomedical Engineering, CAMS & PUMC (Chinese Academy of Medical Sciences and Peking Union Medical College), Tianjin 300192, China

**Keywords:** electrical impedance tomography, ultrasound tomography, dual-modality imaging, lagrange-newton method

## Abstract

An image reconstruction method is proposed based on Lagrange-Newton method for electrical impedance tomography (EIT) and ultrasound tomography (UT) dual-modality imaging. Since the change in conductivity distribution is usually accompanied with the change in acoustic impedance distribution, the reconstruction targets of EIT and UT are unified to the conductivity difference using the same mesh model. Some background medium distribution information obtained from ultrasound transmission and reflection measurements can be used to construct a hard constraint about the conductivity difference distribution. Then, the EIT/UT dual-modality inverse problem is constructed by an equality constraint equation, and the Lagrange multiplier method combining Newton-Raphson iteration is used to solve the EIT/UT dual-modality inverse problem. The numerical and experimental results show that the proposed dual-modality image reconstruction method has a better performance than the single-modality EIT method and is more robust to the measurement noise.

## 1. Introduction

Electrical impedance tomography (EIT) is a non-invasive technique to reconstruct the distribution of the media within a closed vessel based on the differences of their conductivities. Due to its advantages of high speed, non-radiation, and low cost, EIT has been widely researched and used in many fields, such as, industrial process imaging [1,2], medical imaging [3,4], geological exploration [5,6], structural health monitoring [7,8], and nondestructive evaluation [9,10]. However, as a soft-field imaging technique, EIT usually suffers from the low spatial resolution and imaging accuracy for its inherent nonlinearity and ill-posedness [11]. It has hindered the further application of EIT in many fields, especially for medical imaging, where a higher imaging accuracy is usually expected [12]. Thus, the study of improving the EIT spatial resolution and imaging accuracy can always attract the attention of the researchers.

Many researchers have devoted to the study of improving the spatial resolution and imaging accuracy of EIT and many methods have been developed. For the nonlinearity of EIT, the directly linearization [13], iterative linearization [14], and directly nonlinear methods [15] have been proposed to formulate the EIT inverse problem. For the ill-posedness of EIT, the regularization methods, such as, Tikhonov regularization [14], Total Variation (TV) regularization [16], and sparse regularization [17], have been proposed to constrain the solution space of EIT inverse problem by introducing the prior information about the targets to be reconstructed. Thus, the construction of the prior information about the target is important for the EIT inverse problem. The prior information is usually predetermined and added into the EIT inverse problem. A commonly used method for constructing the prior information is to use the characteristics of the target itself. For example, in the imaging problems of small targets, the sparse prior constraints are commonly used [17]. For specific target imaging problems, the statistical modeling methods can be used to extract the similarity information of the targets, such as structure or shape, and construct the statistical prior information about the targets. For example, in EIT lung function monitoring, the statistical shape prior extracted from computed tomography (CT) images can be used to alleviate the ill-posedness of EIT inverse problem and improve the EIT imaging accuracy and measurement noise robustness [18].

Although the spatial resolution and imaging accuracy have been improved to some extent by the regularization constraint from the prior information in EIT inverse problem, the acquisition of the prior information about the target usually falls into the problems of lack of the data sources and feature extraction difficulties. In addition, the individual difference of the imaging targets can also invalidate the prior information in some practical applications. Thus, relying on a single-modality imaging technique is difficult to substantially improve the EIT spatial resolution and imaging accuracy because the available prior information is usually limited. Considering the potential information complementarity between different imaging modalities, the dual-modality imaging techniques have attracted more and more attention in the past decade. For example, the positron emission computed tomography (PET)/CT [19], magnetic resonance imaging (MRI)/CT [20] and Ultrasound/MRI [21] have been successfully used in medical diagnosis. The ultrasound tomography (UT)/electrical capacitance tomography (ECT) [22], CT/ECT [23], Gamma Densitometry Tomography (GDT)/EIT [24] have been widely used in multiphase flow process parameters detection. Thus, the dual-modality imaging technique is promising to improve the EIT imaging accuracy by adding the prior information from the other imaging modality.

UT is also a non-invasive technique to reconstruct the media distribution based on the differences of their acoustic impedances and has been widely used in industrial imaging [25] and medical imaging [26]. Different from the EIT, UT is a ‘hard-field’ imaging technique and the spatial resolution and imaging accuracy of UT is mainly depended on the amount of measured data available. The more measured data means that more ultrasonic transducers are needed in UT [27]. However, the number of the ultrasonic transducer is usually not enough compared to the need for the imaging accuracy in practical applications. Fortunately, UT can obtain some accurate local media distribution information even the number of the ultrasonic transducer is limited [28,29]. Thus, the prior information about the media distribution from UT is promising to improve the imaging accuracy of EIT.

Some studies have been reported about the EIT/UT dual-modality imaging in the past decade. In 2008, Steiner et al. proposed an EIT/ultrasound reflection tomography (URT) dual-modality imaging method for small object detection in biomedical applications, where the measurement information from URT was used to construct the weighted regularization matrix in EIT inverse problem [30]. In 2010, Wan et al. presented a transrectal ultrasound coupled EIT imaging method for transrectal cancer detection, where the contour of the target was captured by ultrasound measurement, and then used to guide the EIT image reconstruction [31]. In 2015, Teniou et al. proposed an electrical resistance tomography (ERT)/URT dual-modality imaging method to detect the small lesions in biological soft tissue, where the measurements from URT was used as hard constraints during the EIT image reconstruction process [32]. In 2017, Pusppanathan et al. proposed an ECT/ultrasound transmission tomography (UTT) dual-modality imaging method for multiphase flow imaging, where the images from the ECT and UTT are fused by the pixel-based fuzzy logic method, and then used to distinguish oil/gas/water three-phase media [33]. In 2018, Liang et al. proposed a shape-based EIT/URT dual-modality imaging method, where some accurate boundaries information about the inclusions detected from URT are used to improve the EIT inclusion boundary reconstruction accuracy [34]. In addition, the emerging impediography can improve the EIT image accuracy by coupling the mechanical effects driven from ultrasound waves [35,36,37]. Although the EIT/UT dual-modality imaging has made some progresses, the way of information fusion between EIT and UT is still an open issue in EIT/UT dual-modality imaging researches.

In this paper, a dual-modality image reconstruction method combining EIT and UT is proposed. Some prior information about the background medium distribution is determined by the ultrasound reflection and transmission measurements, and then used as a hard constraint added into the EIT inverse problem through an equality constraint equation. Then, the ultrasound constraint EIT image reconstruction problem is reformed by the Lagrange multiplier method and iteratively solved with Newton-Raphson method. To test the performance of the proposed dual-modality imaging method, the numerical and experimental tests are conducted.

## 2. Mathematical Model of EIT and UT

### 2.1. EIT Mathematical Model

EIT mathematical model is derived from Maxwell’s electromagnetic theory under the condition of low frequencies. In the quasi-static approximation, the electrical fields can be described in terms of a scalar voltage potential *u* satisfying the following equation:

(1) ∇·σ∇u=0 in Ω,

where *σ* represents the conductivity in the imaging domain Ω.

Considering the contact impedance between the background medium and electrodes, the complete electrode model (CEM) is used to define the boundary conditions [38]:

(2) $$\{\begin{array}{l} \sigma \nabla u\cdot \vec{n}=0,\;\mathrm{on}\;\partial \Omega \backslash \cup_{l=1}^{L}e_{l}\\ \int_{e_{l}}\sigma \nabla u\cdot \vec{n}=I_{l},\\ u+z_{l}\sigma \nabla u\cdot \vec{n}=U_{l},\;\mathrm{on}\;e_{l} \end{array},$$

where ∂Ω is the boundary of the imaging domain Ω, $e_{l}\subset \partial \Omega$ represents the location covered by the *l*-th electrode, *L* is the total number of the electrodes, $\vec{n}$ is the outward normal vector on ∂Ω, $z_{l}$ is the contact impedance between the *l*-th electrode and background medium, $I_{l}$ and $U_{l}$ are the electric current and electric potential at the *l*-th electrode, respectively. Subject to the law of the conservation of charge, the electric current and voltage in the electrodes satisfy the following equation:

(3) $$\sum_{l=1}^{L}I_{l}=0\;\mathrm{and}\;\sum_{l=1}^{L}U_{l}=0,$$

Thus, with the definition of EIT mathematical model and the corresponding boundary conditions, the electric potential, conductivity, electric current, and contact impedance can be formulated as the following nonlinear equation:

(4) U=u(σ,z,I),

Considering a small change on the conductivity, the corresponding change on the boundary voltage can be linearly represented using Equation (4) as follows:

(5) $$\begin{array}{cc} \Delta U & =u\left(\sigma ,z,I\right)-u\left(\sigma_{0},z,I\right)={\frac{du\left(\sigma ,z,I\right)}{d\sigma }|}_{\sigma =\sigma_{0}}\left(\sigma -\sigma_{0}\right)+O\left({\left(\sigma -\sigma_{0}\right)}^{2}\right)\\ & =u^{\prime }\left(\sigma_{0},z,I\right)\Delta \sigma +O\left({\left(\Delta \sigma \right)}^{2}\right) \end{array},$$

If the change of conductivity Δσ is small enough, the high order terms $O\left({\left(\Delta \sigma \right)}^{2}\right)$ can be neglected, and Equation (5) will be simplified as follows:

(6) $$\Delta U=u^{\prime }\left(\sigma_{0},z,I\right)\Delta \sigma ,$$

further, the Equation (6) is usually discretized as follows:

(7) y=Jx,

where $y\in R^{M\times 1}$ represents the boundary voltage difference, $x\in R^{N\times 1}$ represents the unknown conductivity difference, $J\in R^{M\times N}$ represents the Jacobian matrix, which can be calculated using the Geselowitz’s sensitivity theorem [39]:

(8) $$J_{m,n}=-\int_{\Omega_{n}}\frac{\nabla u\left(I^{i}\right)}{I^{i}}\frac{\nabla u\left(I^{j}\right)}{I^{j}}dS,$$

where $J_{m,n}$ is the element at the *m*-th row and *n* column of the Jacobian matrix, $u\left(I^{i}\right)$ is the electric potential for the *i*-th electrode pair with electric current $I^{i}$, $u\left(I^{j}\right)$ is the electric potential for the *j*-th electrode pair with electric current $I^{j}$, $\Omega_{n}$ is the region covered by *n*-th pixel.

For a given conductivity difference *x*, the boundary voltage *y* can be determined by the Equations (1)–(3). This process is usually mentioned as the EIT forward problem, where the Boundary Element Method (BEM) solver developed by Ren et al. is used to calculate the EIT forward problem and obtain the sensitivity matrix by the perturbation method [40,41]. In contrast, given the boundary voltage *y*, the EIT inverse problem is to calculate the conductivity difference *x* by the Equation (7). Since the sensitivity matrix *J* is usually singular, the unknown conductivity difference *x* cannot be directly obtained from the Equation (7) in EIT inverse problem. The regularization methods are commonly used to solve it, which will be introduced in the latter section.

### 2.2. UT Mathematical Model

**Figure 1:**
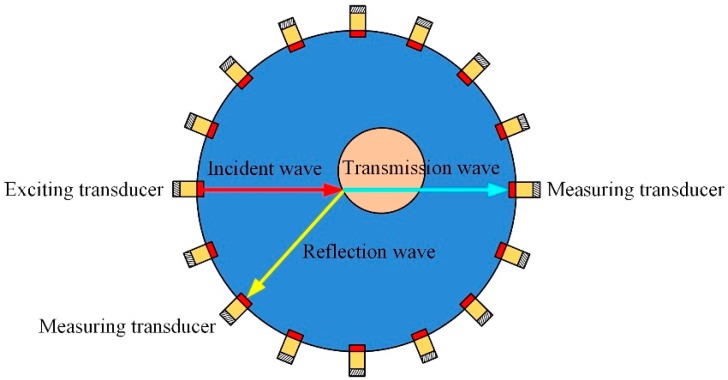
Ultrasound transmission and reflection physical model.

When the influence of medium heterogeneity on acoustic wave propagation is not considered, the propagation of ultrasonic wave in medium can be approximated as linear propagation model. This model is usually referred as the geometrical/ray acoustic model and widely used in UT image reconstruction. Thus, the mathematical model of UT can be referred to the geometrical optics or ray optics model, and the most commonly considered modes of the ultrasonic wave propagation are transmission and reflection modes, as shown in Figure 1. In addition, the piston transducer is used in the paper, where the energy of the ultrasonic wave is mainly concentrated in a narrow main lobe area [42]. The power of the transmission and reflection wave is mainly affected by the acoustic impedance of the media where the ultrasonic wave pass through, and it can be described as follows:

(9) $$\alpha_{R}={\left(\frac{P_{r}}{P_{i}}\right)}^{2}={\left(\frac{Z_{2}-Z_{1}}{Z_{2}+Z_{1}}\right)}^{2},$$

(10) $$\alpha_{T}={\left(\frac{P_{t}}{P_{i}}\right)}^{2}={\left(\frac{2Z_{2}}{Z_{2}+Z_{1}}\right)}^{2},$$

where $P_{i},\;P_{r},\;P_{t}$ represent incident, reflection, and transmission sound pressures, respectively. $\alpha_{R}$ and $\alpha_{T}$ represent the reflection and transmission coefficients, respectively. $Z_{1}$ and $Z_{2}$ are the acoustic impedance of the media and equal to the product of medium density *ρ* and sound velocity *c*.

#### 2.2.1. Transmission Mode Mathematical Model

Under the assumption that the ultrasonic wave is propagating along the straight line, the mathematical model of transmission mode UT is similar to the x-ray CT. The ultrasonic waves sending from the exciting transducer will pass through the media and arrive at the measuring transducers. Consider that the acoustic attenuation effect of the medium, the transmission mode UT usually utilizes the energy attenuation of the ultrasonic waves to invert the medium distribution in the ultrasonic wave propagation paths. Inspired from the Equation (10), we reformulate the sending acoustic energy $I_{e}$ from the exciting transducer and the receiving acoustic energy $I_{m}$ from the measuring transducer as the following equation:

(11) $$\iint_{\Omega_{k}}\mu dS=-\ln\frac{I_{m}}{I_{e}},$$

where *μ* is the medium’s attenuation coefficient, $\Omega_{k}$ is the region where the *k*-th ultrasonic wave pass through.

Consider a scenario that medium located in the region $\Omega_{k}$ changes, the corresponding attenuation coefficient *μ* in $\Omega_{k}$ will be also changing to μ+δμ. Then, the energy attenuation of the ultrasonic wave in the region $\Omega_{k}$ will change and the Equation (11) can be rewritten as follows:

(12) $$\iint_{\Omega_{k}}\left(\delta \mu \right)dS=\iint_{\Omega_{k}}\left(\mu +\delta \mu \right)dS-\iint_{\Omega_{k}}\mu dS=-\ln\frac{I_{m}^{a}}{I_{e}}-\left(-\ln\frac{I_{m}^{b}}{I_{e}}\right)=\ln\frac{I_{m}^{b}}{I_{m}^{a}},$$

where δμ is the variation of the attenuation coefficient in $\Omega_{k}$, $I_{m}^{b}$ and $I_{m}^{a}$ represent the acoustic energy before and after the attenuation coefficient changes, respectively.

#### 2.2.2. Reflection Mode Mathematical Model

Similar to the propagation of optics ray in the medium, the mathematical model of the reflection mode UT can be derived from the Fermat’s principle, where the ultrasonic waves will reflect at the media interface and continue to propagate to the measuring transducer along the shortest propagation path. Thus, the ultrasonic waves sending from the exciting transducer will be reflected at the interface where the acoustic impedances change and continue to propagate in the background medium to the measuring transducer. When the exciting and measuring transducer are determined, the detected points will fall on an elliptical arc. This elliptical arc can be determined by the coordinates of the exciting and measuring transducer and the time of flight (TOF) of the ultrasonic wave, which can be described as follows:

(13) $$\frac{1}{c}\cdot \left[\sqrt{{\left(x_{d}-x_{e}\right)}^{2}+{\left(y_{d}-y_{e}\right)}^{2}}+\sqrt{{\left(x_{d}-x_{m}\right)}^{2}+{\left(y_{d}-y_{m}\right)}^{2}}\right]=t_{f},$$

where *c* is the sound speed in the background medium, $t_{f}$ is the TOF of the ultrasonic wave in the background medium, $\left(x_{d},y_{d}\right)$ is the coordinate of the detected point, which obeys the arc distribution, $\left(x_{e},y_{e}\right)$ and $\left(x_{m},y_{m}\right)$ are the coordinates of the exciting and measuring transducer, respectively.

### 2.3. Complementarity of EIT and URT Sensitive Field

**Figure 2:**
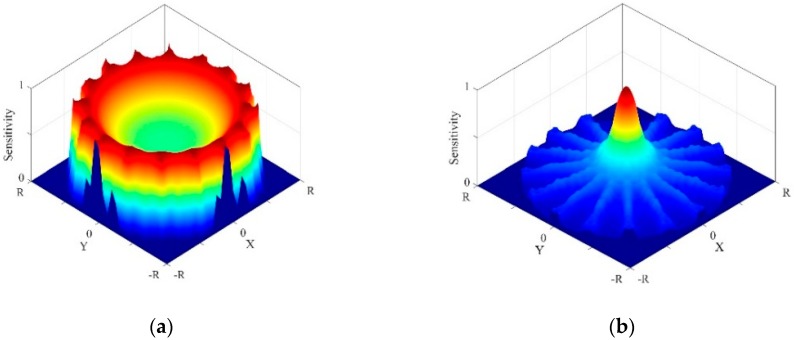
3-D sensitivity distribution: (**a**) Electrical impedance tomography (EIT), (**b**) ultrasound tomography (UT).

The boundary measurements of EIT and UT have different sensitivities about the changes of the sensitive parameters in different spaces of the imaging domain, and higher sensitivity means higher detection resolution and accuracy. In EIT, the electric current is injected into the imaging domain through the electrodes attached at the boundary. The electric field lines near the edge of the imaging domain are dense than the central region of the imaging domain. Therefore, the EIT measurements have a higher sensitivity to the changes in conductivity near the edge of the imaging domain. According to the reciprocity principle, the sensitivity of EIT measurements about the changes of conductivity is proportional to the integration of the electric field intensity [43]. As shown in Figure 2a, EIT has higher sensitivity near the edge of the imaging domain. In UT, the ultrasonic waves emitted by the transducers are mainly concentrated in a narrow main lobe range along the radial of the transducer. Only the changes of acoustic impedance occurring at the propagation path of ultrasonic waves will cause the change on reflection or transmission measurements. In the imaging domain, all the ultrasonic waves propagation paths of the transducers are intersected in the central of the imaging domain. Therefore, the UT measurements have a higher sensitivity about the changes in acoustic impedance near the central of the imaging domain [44]. According to the radon transform theory, the sensitivity of UT measurements about the change of acoustic impedance can be formed by the superimposition of all single transducer ultrasonic waves propagation paths. As shown in Figure 2b, UT has higher sensitivity near the central of the imaging domain. Based on the complementarity of EIT and UT sensitivity space distribution, the combination of EIT and UT is expected to improve the imaging resolution and accuracy.

## 3. EIT/UT Dual-Modality Image Reconstruction

### 3.1. Unification of Mesh Model and Reconstruction Target

**Figure 3:**
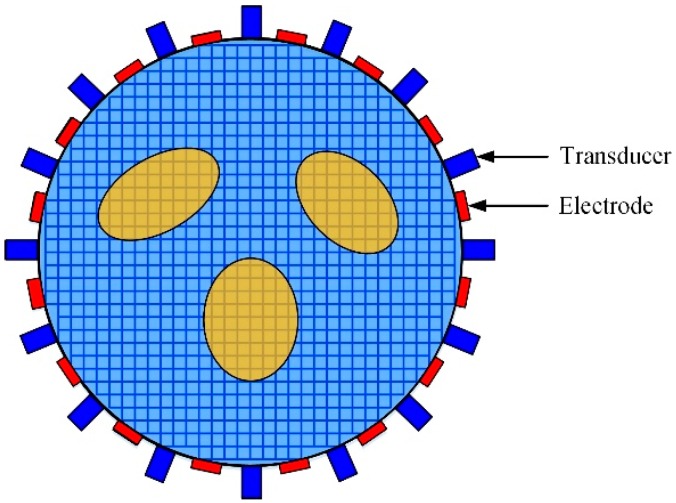
Mesh model of EIT/UT dual-modality imaging.

In the paper, the purpose of both EIT and UT is to reconstruct the media distribution based on the change of conductivity or acoustic impedance distribution. In some applications, when the media distribution changes, the conductivity and acoustic impedance will also change. For example, in gas/liquid two-phase flow cross-section reconstruction problem, the presence of bubble will change the distribution of conductivity and acoustic impedance, the targets of EIT and UT are all the reconstruction of the bubble distribution. Thus, the reconstruction target of EIT and UT can be uniformed to the changes of the media distribution, which means that the background medium has the same representation in EIT and UT. In addition, another factor to consider is the mesh model. Based on this demand of reconstructing the same unknown media distribution using EIT and UT, the mesh model for EIT and UT should be also uniformed in the same imaging region. To achieve the purpose, the same mesh model is used in EIT and UT, as shown in Figure 3.

### 3.2. Total Variation Regularization for EIT

It’s known that EIT inverse problem is a typical ill-posed inverse problem due to the large condition number of the Jacobian matrix *J*, which means that it’s unrealistic to directly solve the unknown *x* from the Equation (7). To overcome this problem, the regularization methods are usually adopted. First, we convert the EIT inverse problem to find the minimum from the least-square equation ${\left\|Jx-y\right\|}_{2}^{2}$. Then, a penalty term, also referred as regularization term, is added to stabilize the solution process by constraining the solution space. This process can be formulated in the following equations:

(14) $$\underset{x\in R^{N}}{\min}f\left(x\right),$$

(15) $$f\left(x\right)=\frac{1}{2}{\left\|Jx-y\right\|}_{2}^{2}+\beta R\left(x\right),$$

where the regularization parameter *β* is a positive constant and balances the residual term ${\left\|Jx-y\right\|}_{2}^{2}$ and the penalty term R(x) in the inverse problem solving process. There are many forms of the regularization term based on the difference of the solution space constraints. Here, the Total Variation (TV) regularization term is adopted, and the regularization term can be written as:

(16) $$R\left(x\right)=\int_{\Omega }\left|\nabla x\right|dS,$$

However, the Equations (14)–(16) cannot be directly solved due to the non-differentiability of |∇x|. To overcome this problem, a smooth approximation is usually adopted [45]. Then, the regularization term R(x) can be rewritten as:

(17) $$R\left(x\right)=\int_{\Omega }\sqrt{{\left|\nabla x\right|}^{2}+\theta }dS,$$

where θ>0 is a predetermined small constant and is selected as $1\times 10^{-6}$ here.

### 3.3. Constraints Information from UT Measurements

As mentioned above, the purpose of EIT and UT is to reconstruct the media distribution based on the change of conductivity or acoustic impedance from the boundary measurements. Since there is no change in the conductivity and acoustic impedance in the background medium region, the image reconstruction of the background medium satisfies the following relationship in EIT and UT:

(18) $$\Omega^{b}=\Omega_{EIT}^{b}\left(\delta \sigma =0\right)=\Omega_{UT}^{b}\left(\delta Z=0,\;\delta \mu =0\right),$$

where $\Omega^{b}$ represents the background medium region, $\Omega_{EIT}^{b}$ and $\Omega_{UT}^{b}$ are the background medium region reconstructed from EIT and UT, respectively.

**Figure 4:**
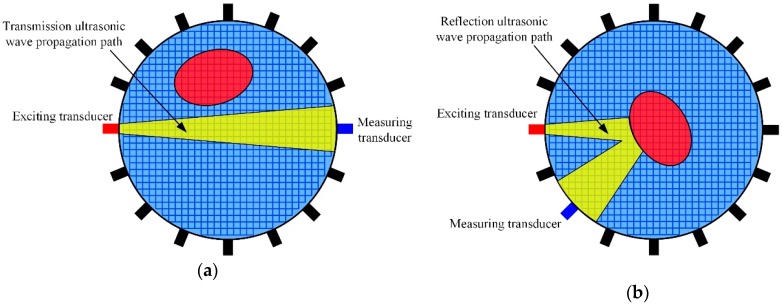
Schematic diagram of ultrasonic wave propagation path: (**a**) there is no target on the ultrasonic waves propagation path, (**b**) there is a target on the ultrasonic waves propagation path.

Thus, the background medium region reconstructed from UT can be used as the prior information in EIT image reconstruction. Consider two scenarios whether there is a target on the ultrasonic wave’s propagation path, as shown in Figure 4a,b. If there is no target on the ultrasonic wave propagation path, the ultrasonic waves sending from the exciting transducer will continue to propagate to the measuring transducer and the attenuation of the acoustic energy is only caused by the background medium. And then, we can mark the pixels on the transmission ultrasonic waves propagation path as background medium region $\Omega_{UT}^{b}$. In contrast, if there is a target on the ultrasonic wave propagation path, the ultrasonic waves sending from the exciting transducer will reflect on the target interface, and then continue to propagate to the measuring transducer through the background medium. The time of flight $t_{f}$ can be extracted from the reflection waves. And then, using the Equation (13) and the main lobe area of the transducer, we can calculate the region covered by the reflection ultrasonic waves propagation paths. After that, we can also mark the pixels on the reflection ultrasonic waves propagation path as background medium region $\Omega_{UT}^{b}$.

Since the conductivity of the background medium region does not change, we can derive the following equation:

(19) h(x)=C x=0,

where h(x) represents the constraint function of UT measurements with respect to the conductivity difference *x*, *C* is the corresponding extend constraint matrix, and it’s calculated by the analytical method:

(20) $$C\left(i,j\right)=\{\begin{array}{l} 1,\;i=1,\ldots ,M_{U},\;j=n\left(i\right)\\ 0,\;\mathrm{otherwise} \end{array},$$

where $C\in R^{M_{U}\times N}$, $M_{U}$ is the total number of pixels in region of $\Omega_{UT}^{b}$, n(i) is the serial number of the pixels located at $\Omega_{UT}^{b}$ in the set of all pixels.

### 3.4. Lagrange-Newton Solution for Dual-Modality Inverse Problem

In summary, we can convert the media distribution image reconstruction problem into the conductivity change image reconstruction problem for the EIT/UT dual-modality imaging. The EIT/UT dual-modality inverse problem can be described as follows:

(21) $$\begin{array}{l} \underset{x\in R^{N}}{\min}f\left(x\right)\\ s.\;t.\;h\left(x\right)=0 \end{array}$$

where *f*(*x*) and *h*(*x*) are defined by Equations (15) and (19), respectively.

Obviously, the problem (21) is a typical equality constraint optimization problem. The Lagrange function combining with Newton method is commonly used to solve it. First, we formulate the following Lagrange function:

(22) $$\Psi \left(x,\lambda \right)=f\left(x\right)-\lambda^{T}h\left(x\right)=\frac{1}{2}{\left\|J\;x-y\right\|}_{2}^{2}+\beta R\left(x\right)-\lambda^{T}h\left(x\right),$$

where *λ* is the Lagrange multiplier. The stability point of the above Lagrange function corresponds to the optimal solution of the unknown *x*, and satisfies the following conditions:

(23) $$\{\begin{array}{l} \frac{\partial \Psi }{\partial x}=\nabla f\left(x\right)-\nabla h{\left(x\right)}^{T}\lambda =0\\ \frac{\partial \Psi }{\partial \lambda }=-h\left(x\right)=0 \end{array},$$

Then, we solve the problem (22) using the Newton-Raphson method, and the iteration solution can be described as $x_{k+1}=x_{k}+{\left(\delta x\right)}_{k}$, where the iteration step length ${\left(\delta x\right)}_{k}$ and the Lagrange multiplier ${\left(\delta \lambda \right)}_{k}$ satisfy the following equation:

(24) $$\left(\begin{array}{cc} W\left(x_{k},\lambda_{k}\right) & -\nabla h{\left(x_{k}\right)}^{T}\\ -\nabla h\left(x_{k}\right) & 0 \end{array}\right)\;\left(\begin{array}{c} {\left(\delta x\right)}_{k}\\ \left(\delta \lambda \right){}_{k} \end{array}\right)=-\left(\begin{array}{c} \nabla f\left(x_{k}\right)-\nabla h{\left(x_{k}\right)}^{T}\lambda_{k}\\ -h\left(x_{k}\right) \end{array}\right),$$

where

(25) $$\{\begin{array}{ccc} \nabla h\left(x\right) & = & \nabla \left(Cx\right)=C\\ \nabla f\left(x\right) & = & \nabla \left(\frac{1}{2}{\left\|Jx-y\right\|}_{2}^{2}+\beta R\left(x\right)\right)=J^{T}\left(Jx-y\right)+\beta L_{\theta }\left(x\right)x\\ W\left(x,\lambda \right) & = & \nabla^{2}\left(\Psi \left(x,\lambda \right)\right)=\nabla^{2}\left(f\left(x\right)-\lambda^{T}h\left(x\right)\right)=J^{T}J+\beta L_{\theta }\left(x\right)\\ L_{\theta }\left(x\right) & = & L_{a}^{T}\left(x\right)H^{-1}\left(x\right)L_{a}\left(x\right), \end{array},$$

where $J^{T}$ is the transpose of *J*, $L_{a}\in R^{Q\times N}$ is a sparse matrix, which can be determined by the topological relationship between the pixels, referring to the literature [45], $H\in R^{Q\times Q}$ is a diagonal matrix whose diagonal entry can be described as follows:

(26) $$H_{qq}\left(x\right)=\sqrt{{\left\|L_{a,q}\left(x\right)x\right\|}^{2}+\theta },\;q=1,2,\ldots ,Q,$$

where $L_{a,q}\in R^{1\times N}$ is the *q*-th row element of $L_{a}$, *Q* is the total number of adjacent element pairs along the horizontal or vertical direction, referring to the literature [45].

To analyze the iteration convergence and determine the iteration termination condition of the problem (21), we define the following penalty function:

(27) $$G\left(x,\lambda \right)={\left\|\nabla f\left(x\right)-\nabla h{\left(x\right)}^{T}\lambda \right\|}_{2}^{2}+{\left\|h\left(x\right)\right\|}_{2}^{2},$$

In summary, the implement of the proposed EIT/UT dual-modality image reconstruction method can be described as Algorithm 1.

**Algorithm 1:** Lagrange-Newton Method for EIT/UT Dual-Modality Image Reconstruction **Step 1: Initialization.**  Calculate the initial value of *x* using linear back-projection (LBP) algorithm [13]: $x_{0}=J^{T}y$,  Given the value of the iteration termination parameter: $\varepsilon \geq 0,\;\varepsilon =1\times 10^{-6}$,  Given the value of the intermediate parameter in iteration: κ∈(0, 1), κ=0.5, η=1. **Step 2: Termination Condition Judgment.**  Calculate $G\left(x_{k},\;\lambda_{k}\right)$ by Equation (27), and judge:  If $G\left(x_{k},\;\lambda_{k}\right)\leq \varepsilon$, stop iteration and return the $x_{k}$ as the optimal estimation value,  Otherwise, calculate ${\left(\delta x\right)}_{k}$ and $\left(\delta \lambda \right){}_{k}$ by the Equation (24). **Step 3: Compute Step Length.**  Calculate $G\left(x_{k}+\eta {\left(\delta x\right)}_{k},\lambda_{k}+\eta {\left(\delta \lambda \right)}_{k}\right)$ and $G\left(x_{k},\;\lambda_{k}\right)$ by Equation (27), and judge:  If $G\left(x_{k}+\eta {\left(\delta x\right)}_{k},\lambda_{k}+\eta {\left(\delta \lambda \right)}_{k}\right)\leq \left(1-\kappa \eta \right)G\left(x_{k},\lambda_{k}\right)$, go to Step 4,  Otherwise, set η=η/4, continue to the Step 3. **Step 4: Iteration Update.**  Update the conductivity difference *x*: $x_{k+1}=x_{k}+\eta {\left(\delta x\right)}_{k}$,  Update the Lagrange multiplier *λ*: $\lambda_{k+1}=\lambda_{k}+\eta {\left(\delta \lambda \right)}_{k}$,  Set *k*=*k*+1 and go to Step 2.


## 4. Results

To evaluate the performance of the proposed EIT/UT dual-modality imaging method, a series of numerical and experimental tests are carried out and compared with single-modality EIT method. In the tests, three typical EIT image reconstruction methods, Newton’s One-Step Error Reconstructor (NOSER), L1 regularization, and TV regularization, are used in single-modality EIT image reconstruction. Considering that the selection of regularization parameters has a great influence on the imaging results, the regularization parameters in all methods are empirically selected in the range of $10^{-6}$ to $10^{-3}$, and the one that reached the most similar result to the real model was used in the comparison [45]. In EIT, the adjacent electric current excitation and adjacent voltage measurement model is used to obtain the boundary measurements. In UT, the piston transducers are used in numerical and experimental tests, and the acoustic energy can be better concentrated in a narrow beam range. In transmission mode, three transducers directly opposite to the exciting transducer are used to obtain the transmission measurements. In reflection mode, three transducers on each adjacent side of the exciting transducer are used to obtain the reflection measurements. The ultrasound frequency is 1MHz in the numerical and experimental tests. In addition, the reconstruction results from EIT/UT dual-modality method is simplified as UET in the paper.

### 4.1. Numerical Results and Discussion

The numerical tests are conducted by the commercial multi-physical coupling software COMSOL and MATLAB. The simulation model is designed by simulating the discrete bubbles in a conductive liquid. The conductivity of the bubble is $1\times 10^{-6}$ Sm^−1^ and the background medium conductivity is $1\times 10^{-2}$ Sm^−1^. The density of the bubble is 1.29 kgm^−3^ and sound velocity is 340 ms^−1^. The density of the background medium is 1000 kgm^−3^ and sound velocity is 1400 ms^−1^. The radius of the imaging domain is 75 mm, and 16 electrodes and 16 ultrasound transducers are staggered evenly and arranged on the periphery of the observation domain, as shown in Figure 3. The piston ultrasound transducers sequentially send and receive the three periods sinusoidal pulse signal in the narrow field range.

#### 4.1.1. Quantitative Index

To evaluate the reconstruction results, four quantitative indexes of relative image error (*RE*), correlation coefficient (*CC*), position error (*PE*) and area difference (*AD*), are used in the paper. The *RE* and *CC* are usually used to describe the degree of difference of the true conductivity difference and reconstructed conductivity difference. The definition of *RE* and *CC* can be expressed as follows:

(28) $$RE=\frac{{\left\|x-x^{*}\right\|}_{2}}{{\left\|x^{*}\right\|}_{2}},$$

(29) $$CC=\frac{\sum_{n=1}^{N}\left(x_{n}-\bar{x}\right)\left(x_{n}^{*}-{\bar{x}}^{*}\right)}{\sqrt{\sum_{n=1}^{N}{\left(x_{n}-\bar{x}\right)}^{2}\sum_{n=1}^{N}{\left(x_{n}^{*}-{\bar{x}}^{*}\right)}^{2}}},$$

where *x* is the calculated conductivity difference vector, $x^{*}$ is the real conductivity difference vector, $x_{n}$ and $x_{n}^{*}$ are the *n*-th element of *x* and $x^{*}$, respectively, $\bar{x}$ and ${\bar{x}}^{*}$ are the mean values of *x* and $x^{*}$, respectively. A lower value of *RE* and a higher value of *CC* mean a good image reconstruction result.

The *PE* and *AD* are respectively used to evaluate the target position and shape reconstructed accuracy by comparing the segmented reconstructed images and the real distribution images. The segmented threshold is selected as 25% of the minimum pixel amplitudes, as follows:

(30) $${\left[x_{q}\right]}_{n}=\{\begin{array}{l} 1,\;\mathrm{if}\;{\left[x\right]}_{n}\leq \frac{1}{4}\min\left(x\right)\\ 0,\;\mathrm{otherwise} \end{array},$$

where *x* is the calculated image pixel amplitudes (conductivity difference), $x_{q}$ is the segmented image pixel amplitudes. The *PE* and *AD* are defined as follows:

(31) $$PE=\left|c_{t}-c_{q}\right|,$$

(32) $$AD=\frac{\mathrm{sum}\left(x_{b}\cup x_{q}\right)-\mathrm{sum}\left(x_{b}\cap x_{q}\right)}{N},$$

where $c_{t}$ is the center of gravity of the target in real conductivity difference distribution image, $c_{q}$ is the center of gravity of the target in reconstructed conductivity difference distribution image, $x_{b}$ is the binary image of the real conductivity difference distribution, $x_{q}$ is the segmented image of the reconstructed conductivity difference distribution image, *N* is the total number of the pixels.

#### 4.1.2. Image Reconstruction Results Analysis

**Figure 5:**
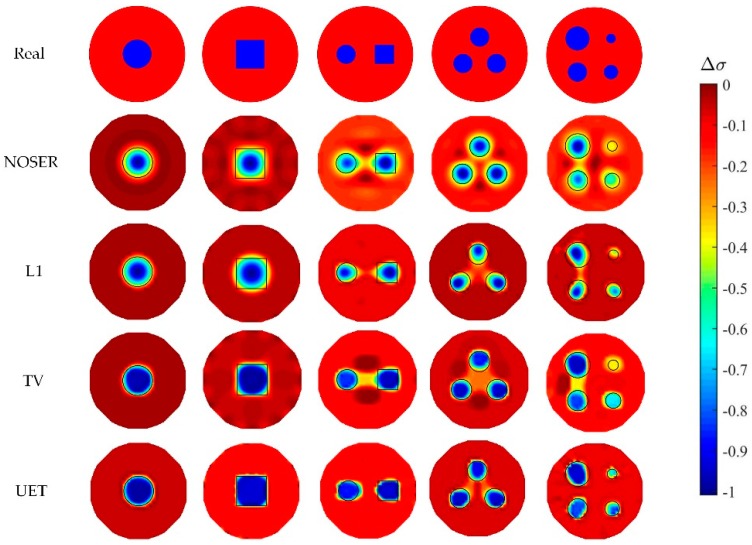
Reconstruction results with no noise.

**Figure 6:**
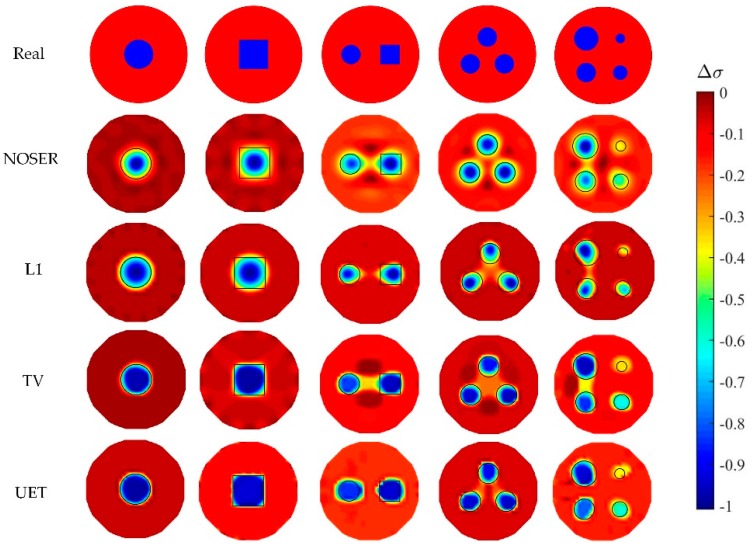
Reconstruction results with 40 dB noise.

**Figure 7:**
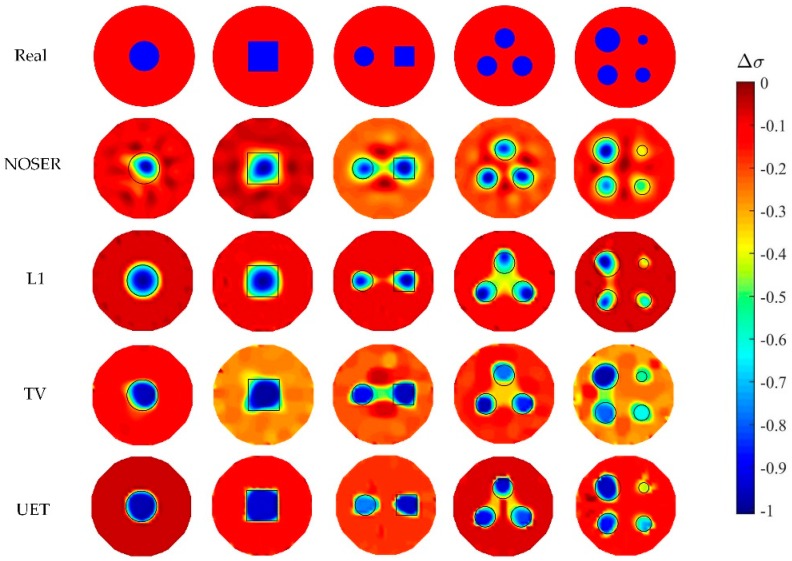
Reconstruction results with 25 dB noise.

**Figure 8:**
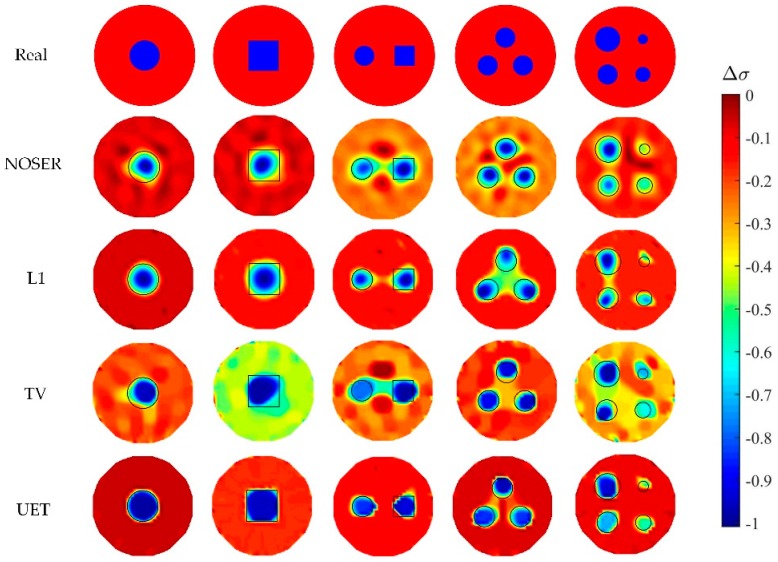
Reconstruction results with 20 dB noise.

**Figure 9:**
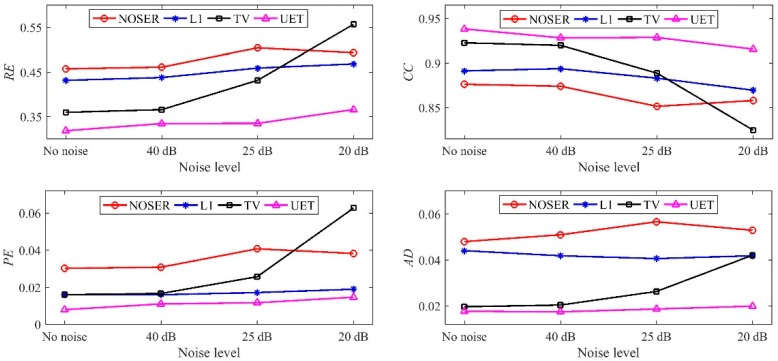
Mean value of the quantitative indexes.

In numerical tests, five different models are used to compare the proposed EIT/UT dual-modality imaging method (UET) and the single-modality EIT imaging method using NOSER, L1, TV. The image reconstruction results are shown in Figure 5, Figure 6, Figure 7 and Figure 8 when adding no noise, 40 dB, 25 dB, 20 dB Gaussian noise in EIT measurements, respectively. The real targets boundaries are indicated by the solid black line in the reconstructed images. It can be seen from the Figure 5, Figure 6, Figure 7 and Figure 8 that the UET has a cleaner background and better imaging performance compared with the other methods, which can be also verified by the quantitative indexes, as shown in Figure 9. For different quantitative indexes, we respectively calculate their mean values in five media distribution models under a certain noise condition. When the noise level increases, the imaging results from single-modality EIT using NOSER, L1 and TV are gradually getting worse, while the proposed UET method can always better reconstruct the media distribution in different noise levels. Thus, the proposed UET method is more robust to the EIT measurement noise. In addition, the expected time of UET is about 0.32 s for per image reconstruction. The expected time of EIT using NOSER, L1, TV are about 0.02 s, 0.2 s, 0.3 s for per image reconstruction, respectively. Thus, the imaging speed of UET is comparable to that of single-modality EIT using TV method, but the imaging accuracy of UET have an obvious improvement.

#### 4.1.3. Comparison of Edge Reconstruction Ability

**Figure 10:**
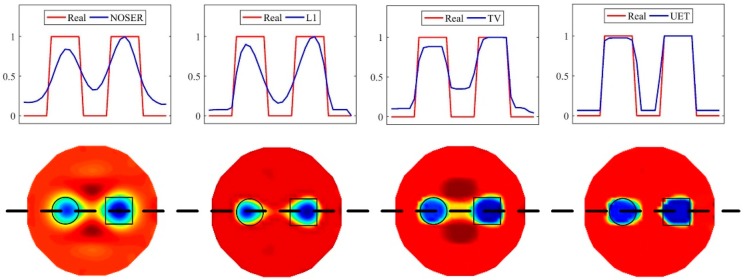
Contour of the reconstructed images along the center horizontal line.

It can be seen from the numerical results that the UET has a cleaner edge than other methods. To verify this conclusion, we further analyze the numerical results of the model with one circle and one square in Figure 5. First, the pixel values of the reconstructed images are normalized to the interval [0, 1]. Then, the contour of the pixel values along the center horizontal line of the reconstructed images is drawn in Figure 10. It’s obvious that the contour of UET reconstructed image is closer to the real contour. Thus, the proposed UET method has a better edge reconstruction ability than the single-modality EIT methods.

### 4.2. Experimental Results and Discussion

**Figure 11:**
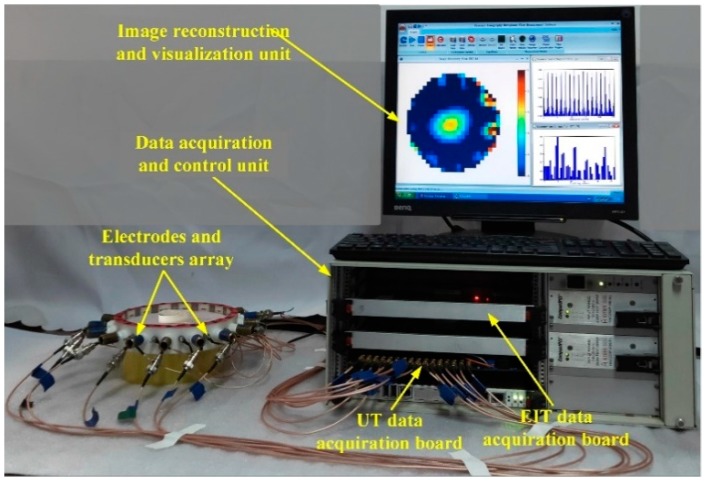
EIT/UT dual-modality data acquisition system.

To further verify the performance of the proposed method, a set of experimental tests was carried out. The experimental system is shown in Figure 11, which is developed by Tianjin University, China [46]. It mainly contains three parts, the sensor array, the data acquisition and control unit, and the image reconstruction and visualization unit. The radius of the sensor is 75 mm and 16 electrodes and 16 ultrasonic transducers are evenly placed on the periphery of the sensor. The contact resistance between the electrodes and background medium is 37.1 Ω/cm^2^. The background medium consists of Na_2_SO_4_ solution with the conductivity of 1.41 mS/cm. The data acquisition speed of EIT is about 1000-frames/s, and the data acquisition speed of UT is about 17-frames/s. The expected time of the image reconstruction in experimental tests is similar to numerical tests.

**Figure 12:**
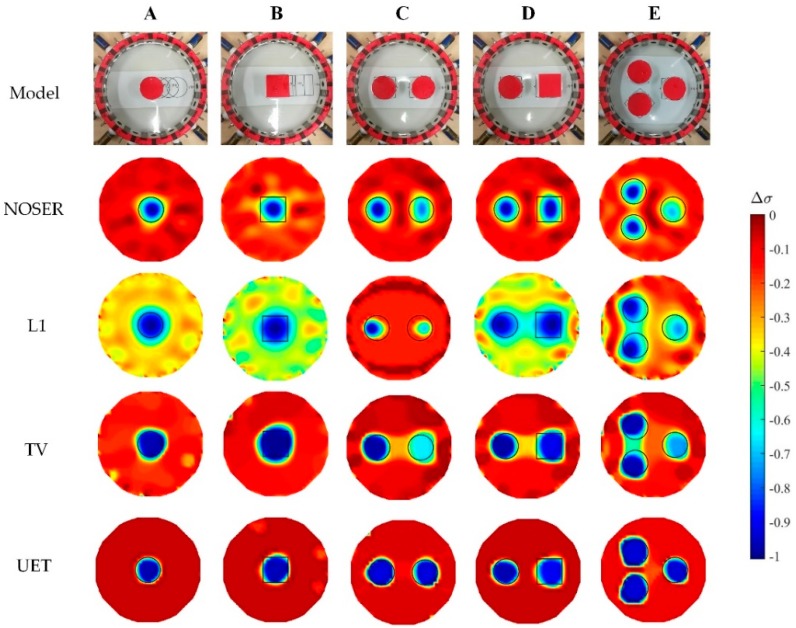
Reconstruction results of the experimental tests.

**Figure 13:**
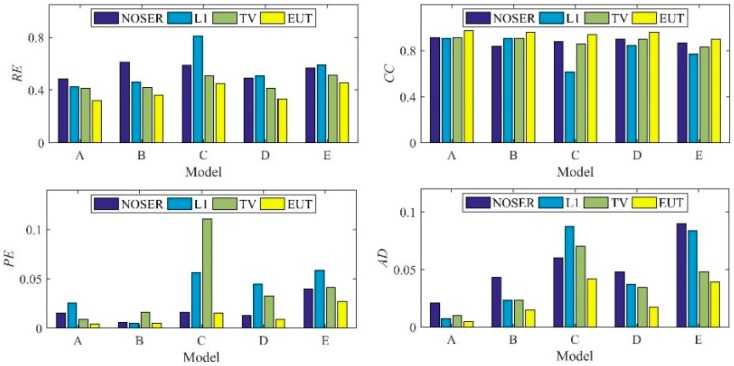
Quantitative analysis of the experimental results.

The experimental results are shown in Figure 12, where five different distribution models (A, B, C, D, E) of the nylon rod ($10^{-11}$ mS/cm) are used. It can be seen from the results that the proposed UET method has a better imaging performance compared with the single-modality EIT methods. The quantitative analysis of the experimental results is shown in Figure 13, which can also verify the conclusion that the proposed UET method shows better reconstruction performance.

## 5. Conclusions

A novel EIT/UT dual-modality imaging method is proposed, where the measurement information obtained from UT can be used as prior constraint and improve the EIT image reconstruction performance. The dual-modality inverse problem is constructed by the equality constraint equation, and then, solved by the Lagrange-Newton method. The numerical and experimental tests show that the proposed EIT/UT dual-modality imaging method has a better image reconstruction performance than the traditional single-modality EIT methods, where the reconstructed images of UET have clearer targets edges and less artifacts in the background region. In the experimental tests, the average *RE* of UET have reduced 29.7%, 31.1%, 14.9% than single-modality EIT using NOSER, L1, TV, respectively. The average *CC* of UET have improved 7.7%, 16.9%, 7.4% than single-modality EIT using NOSER, L1, TV, respectively. The average *PE* of UET have reduced 32.9%, 68.2%, 71.3% than single-modality EIT using NOSER, L1, TV, respectively. The average *AD* of UET have reduced 55.1%, 50.6%, 36.5% than single-modality EIT using NOSER, L1, TV, respectively. In addition, the UET shows better performance of edge reconstruction, where the contour of UET reconstructed image is closer to the real contour than single-modality EIT methods. Furthermore, the proposed UET method is more robust to the EIT measurement noise, where the quantitative indexes of UET have no significant change with the increase of the measurement noise. The future work will focus on the research of enriching the UT measurements by adding the number of the transducers or increasing the diffusion angle of the transducers, and further improve the image reconstruction performance of EIT/UT dual-modality imaging.
